# Synergistic Effect of miR-200 and Young Extracellular Matrix-based Bio-scaffolds to Reduce Signs of Aging in Senescent Fibroblasts

**DOI:** 10.1007/s12015-022-10438-5

**Published:** 2022-08-27

**Authors:** Georgia Pennarossa, Teresina De Iorio, Sharon Arcuri, Fulvio Gandolfi, Tiziana A. L. Brevini

**Affiliations:** 1 Laboratory of Biomedical Embryology, Department of Veterinary Medicine and Animal Sciences, Center for Stem Cell Research, Università degli Studi di Milano, 20134 Milan, Italy; 2 Laboratory of Biomedical Embryology, Department of Agricultural and Environmental Sciences - Production, Landscape, Agroenergy, Università degli Studi di Milano, Milan, 20133 Italy

**Keywords:** Aging, Cellular rejuvenation, ECM-based bio-scaffolds, micro-RNA, miR-200 family, Cell senescence

## Abstract

Aging is defined as a complex, multifaceted degenerative process that causes a gradual decline of physiological functions and a rising mortality risk with time. Stopping senescence or even rejuvenating the body represent one of the long-standing human dreams. Somatic cell nuclear transfer as well as cell reprogramming have suggested the possibility to slow or even reverse signs of aging. We exploited miR-200 family ability to induce a transient high plasticity state in human skin fibroblasts isolated from old individuals and we investigated whether this ameliorates cellular and physiological hallmarks of senescence. In addition, based on the assumption that extracellular matrix (ECM) provides biomechanical stimuli directly influencing cell behavior, we examine whether ECM-based bio-scaffolds, obtained from decellularized ovaries of young swine, stably maintain the rejuvenated phenotype acquired by cells after miR-200 exposure. The results show the existence of multiple factors that cooperate to control a unique program, driving the cell clock. In particular, miR-200 family directly regulates the molecular mechanisms erasing cell senescence. However, this effect is transient, reversible, and quickly lost. On the other hand, the use of an adequate young microenvironment stabilizes the miR-200-mediated rejuvenating effects, suggesting that synergistic interactions occur among molecular effectors and ECM-derived biomechanical stimuli. The model here described is a useful tool to better characterize these complex regulations and to finely dissect the multiple and concurring biochemical and biomechanical cues driving the cell biological clock.

**Graphical Abstract:**

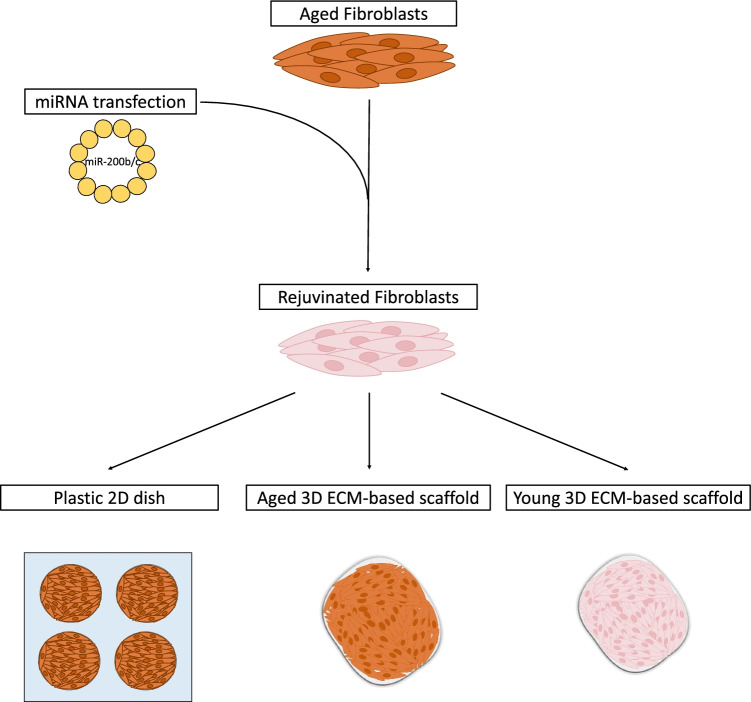

## Introduction

Advances in medical care, improvements in sanitation, and rising living standards contribute to increase life expectancy. Although this reflects positive human development, it also poses new important challenges that will aggravate in the years to come. Among them, aging is one of the most critical and emerging problems in public health, due to the exponential increase of elderly patients suffering with chronic age-onset diseases, often with multiple co-morbidities. Aging is indeed defined as a complex, multifaceted process characterized by a progressive accumulation of macroscopic and microscopic modifications that are accompanied by molecular and cellular damages, negatively affecting organ, tissue, cell, and subcellular organelle functions [1, 2]. These senescence processes are influenced by environmental factors as well as by genetic and epigenetic dysregulations that induce a gradual organism decline [3]. In agreement with this, several studies have described a link between DNA methylation and aging [4–7], demonstrating that around 29% of the methylation sites in the genome is modified with increasing age, thus reflecting the natural development of age-related phenotypes and diseases, such as diabetes, autoimmune and cardiovascular disorders [8].

The possibility to reverse aging hallmarks was suggested, for the first time, by the generation of sheep and mouse offspring with a normal lifespan, after adult somatic cell nuclear transfer (SCNT) [9–11]. Subsequently, the induced pluripotent stem cell (iPSC) technique [12] was used to reprogram senescent and centenarian cells and re-differentiate them into fully rejuvenated cells [13]. During the last years, parallel studies reported the possibility to induce a transient high plasticity state by using a brief exposure to the demethylating agent 5-azacytidine (5-aza-CR) [14–24]. Interestingly, 5-aza-CR treated cells displayed a significant decrease in global DNA methylation, paralleled by an increment in formylcytosine (5fC) and 5-carboxylcytosine (5caC), thus indicating that 5-aza-CR induces both direct and indirect demethylating events [18]. Direct demethylation is known to be driven by the ten-eleven translocation (TET) enzyme family enzymes that play a crucial role during cellular reprogramming, removing the epigenetic blocks and re-activating previously silenced genes [25–28]. Recent studies demonstrated that TET-mediated activation of the miR-200 family promotes a transient high plasticity state and helps fibroblasts to overcome the mesenchymal-epithelial transition (MET) barrier, facilitating iPSC generation [29]. Consistent with this, all members of miR-200 family, namely miR-200a, miR-200b, miR-200c, miR-141 and miR-429, are highly expressed in both ESCs and iPSCs, thus suggesting their involvement in pluripotency induction and maintenance [29].

In the present work, we exploit miR-200 ability to induce a transient high plasticity state in cells isolated from old individuals. We investigate whether this is able to ameliorate cellular and physiological hallmarks of aging in senescent cells. We examine whether extracellular matrix (ECM)-based scaffolds are able to boost and properly maintain the rejuvenated phenotype acquired by cells after miR-200 exposure. Specifically, we take advantage of acellular scaffold low immunogenicity, repopulating porcine decellularized scaffolds with cellular elements of human origin. In addition, we select the ovary based on its complex microanatomy and heterogenous architecture that may provide different mechanotransducive cues, possibly influencing cell proliferation and metabolism and translating physical forces into the activation of local intracellular pathways.

## Materials and Methods

All reagents were purchased from Thermo Fisher Scientific unless otherwise indicated.

### Ethics Statement

Human cells were purchased from the NIGMS Human Genetic Cell Repository at the Coriell Institute for Medical Research (USA). Ethical approval was not required for this study because it did not involve living animals. All the methods in our study were carried out in accordance with the approved guidelines.

### Culture of Human Fibroblasts Obtained From Young and Aged Individuals

Skin fibroblast cell lines obtained from 29 to 32 years old donors (Young, *n* = 3) and from 82 to 92 years old individuals (Aged, *n* = 3) were purchased from the NIGMS Human Genetic Cell Repository at the Coriell Institute for Medical Research (USA). Cells were grown in fibroblast culture medium (FCM) consisting of Eagle’s Minimum Essential Medium (MEM) supplemented with 15% Foetal Bovine Serum (FBS), 2mM glutamine (Sigma-Aldrich), 1% antibiotic/antimycotic solution (Sigma-Aldrich), and maintained in 5% CO_2_ at 37 °C. All experiments were carried out in triplicates at least three times.

### Fibroblast Exposure to miR-200b/c

Cells were plated at a concentration of 2.1 × 10^4^ cells/cm^2^. After 24 h, they were exposed to 100nM of miR-200b/c (predesigned mirVANA Mimics) using RNAi-MAX in Opti-MEM™ medium, following the manufacturer’s instructions, and incubated in 5% CO_2_ at 37 °C for 48 h.

### Culture of miR-200b/c Treated Fibroblasts Onto 2D Systems

At the end of miRNA exposure, cells were returned to FCM and maintained in standard plastic dishes (2D) for 10 days. Cell morphology was monitored daily using an Eclipse TE200 inverted microscope (Nikon), connected to a Digital Sight camera (Nikon). Cultures were arrested at day 2, 5, 7 and 10 and analysed as described below.

### Culture of miR-200b/c Treated Fibroblasts Onto 3D ECM-based Bio-scaffolds

#### Generation of Young and Aged ECM-based Bio-scaffolds

18 ovaries from 6-month (*n* = 9, Young) and 5 years old (*n* = 9, Aged) swine were collected at the local abattoir and subjected to whole-organ decellularization protocol [2, 30–32]. Briefly, organs were frozen at − 80 °C for at least 24 h, thawed in a 37 °C water bath for 30 min, treated with 0.5% sodium dodecyl sulfate (SDS; Bio-Rad) for 3 h, and immersed overnight in 1% Triton X-100 (Sigma-Aldrich). Ovaries were then washed in deionized water (DI-H_2_O) for 9 h and incubated with 2% deoxycholate (Sigma-Aldrich) for 12 h. At the end of the decellularization protocol, the ovarian bio-scaffold generated were extensively washed in DI-H_2_O for 6 h and sterilized with 70% ethanol and 2% antibiotic/antimycotic solution (Sigma-Aldrich) for 30 min. All the steps described were performed at room temperature using an orbital shaker at 300 rpm. A fragment from each bio-scaffold was collected and analysed for evaluating the efficacy of the decellularization process (data not shown).

#### Seeding and Culture of miR-200b/c Treated Fibroblasts Onto Young and Aged ECM-based Bio-scaffolds

miR-200b/c treated fibroblasts were seeded onto Young and Aged ECM-based bio-scaffolds (3D). Specifically, 15 Young and 15 Aged 7 mm x 1 mm fragments were cut from ovarian cortex and repopulated with 6.9 × 10^6^ cells for each, in 300 µL of FCM and maintained in a 5% CO_2_ incubator at 37 °C. Half medium volume was changed every other day. Cultures were arrested at day 2, 5, 7, and 10, and analysed as described below. 15 cortex fragments obtained from Young ovarian bio-scaffolds were repopulated with untreated Aged fibroblasts and used as controls.

### Cell Growth Curve

Growth curve was assessed by plating 2.5 × 10^4^ cells/cm^2^ in 4-well multidishes (Nunc). Cell number was counted using Hycor KOVA ^TM^ Glasstic ^TM^ (Sentinel Diagnostic) and cell viability was determined by trypan blue dye exclusion assay (Sigma-Aldrich). Each time point was assessed in triplicate.

### Cell Proliferation Index

Cell proliferation index was evaluated by proliferating cell nuclear antigen (PCNA) immuno-staining. Cells were fixed in methanol at -20 °C for 15 min, while paraffin-embedded tissues were treated with 10mM Sodium citrate solution (pH 6) containing 0,05% Tween-20 (Sigma-Aldrich) to unmask antigens. Samples were incubated in blocking solution containing 10% Goat Serum (Sigma-Aldrich) in PBS for 30 min. Primary antibody (1:200, Sigma-Aldrich) was incubated for 1 h, followed by a suitable secondary antibody exposure (1:250, Alexa Fluor™) for 1 h. Nuclei were counterstained with 4′,6-diamidino-2- phenylindole (DAPI, Sigma-Aldrich). Samples were observed under the Eclipse TE200 microscope (Nikon). All steps were performed at room temperature, unless otherwise indicated. The number of immuno-positive cells was counted in 10 randomly selected fields at 100× total magnification. A minimum of 500 cells were scored in three independent replicates. The number of PCNA positive cells was expressed as a percentage of the total cell counted.

### Reactive Oxygen Species (ROS) and β-galactosidase (β-GAL) Activity

ROS and β-GAL activities were analysed using human reactive oxygen species (MyBioSource, MBS166870) and Galactosidase beta ELISA kits (MyBioSource, MBS721441). Samples were sonicated at 20 kHz in ice for 20 min, homogenates were centrifuged at 3000 rpm for 20 min and supernatants were collected. Assays were carried out following the manufacturer’s instructions and, at the end of the procedures, total ROS and β-GAL contents were quantified at 450 nm using a Multiskan FC. Standard curves were designed by plotting the absorbance means (y-axis) against the relative concentrations (x-axis) and the best fit line was determined by regression analyses. Absolute quantifications were then calculated.

### Gene Expression Analysis

Quantitative PCR was performed using CFX96 Real-Time PCR detection system (Bio-Rad Laboratories). Total RNA was extracted using the TaqManGene Expression Cells to Ct kit (Applied Biosystems) and DNase I was added in lysis solutions at 1:100 concentration, as indicated by the manufacturer’s instructions. Target genes were analysed using predesigned primers and probe sets from TaqManGene Expression Assays (Table 1). GAPDH and ACTB were used as reference genes. Gene expression levels were quantified with CFX Manager software (Bio-Rad Laboratories) and here reported with the highest expression set to 1 and the other relative to this.

**Table 1:** List of primers used for quantitative PCR analysis

Gene	Description	Cat.N.
ACTB	Actin, beta	Hs01060665_g1
GAPDH	Glyceraldehyde-3-phosphate dehydrogenase	Hs02786624_g1
NANOG	Nanog Homeobox	Hs02387400_g1
OCT4	POU Class 5 Homeobox 1	Hs00999632_g1
REX1	ZFP42 Zinc Finger Protein	Hs01938187_s1
SOX2	SRY-Box Transcription Factor 2	Hs04234836_s1
MKI67	Marker Of Proliferation Ki-67	Hs04260396_g1
TFAM	Transcription Factor A, Mitochondrial	Hs00273372_s1
ROMO1	Reactive Oxygen Species Modulator 1	Hs00603977_m1
PDHA1	Pyruvate Dehydrogenase E1 Subunit Alpha 1	Hs01049345_g1
COX4I1	Cytochrome C Oxidase Subunit 4I1	Hs00971639_m1
CDKN1(P21)	Cyclin Dependent Kinase Inhibitor 1 A	Hs00355782_m1
CDKN2A (P16)	Cyclin Dependent Kinase Inhibitor 2 A	Hs00923894_m1
TP53 (P53)	Tumor protein p53	Hs01034249_m1

### DNA Quantification

Genomic DNA was extracted from fragments, ranging from 10 to 24 mg, with the PureLink® Genomic DNA Kit (Invitrogen), following the provider’s instructions. DNA concentrations were measured with NanoDrop 8000 and normalized against the previously annotated fragment weights.

### Histological Analysis

Repopulated bio-scaffolds were fixed in 10% buffered formalin for 24 h, dehydrated in graded alcohols, cleared with xylene, and embedded in paraffin. Serial microtome sections (5 μm thick) were cut, dewaxed, rehydrated, and stained with hematoxylin and eosin (H&E, BioOptica).

### Cell Density

Cell number was counted in 15 tissue sections obtained from 3 Young (5 sections for each) and 3 Aged (5 sections for each) ECM-based bio-scaffolds repopulated with miR-200b/c treated fibroblasts as well as from 3 Young bio-scaffolds recellularized with untreated Aged cells (5 sections for each). 5-µm-thick sections were stained with DAPI and observed under an Eclipse E600 microscope (Nikon) equipped with a digital camera (Nikon). Pictures were acquired with NIS-Elements Software (Version 4.6; Nikon), using constant exposure parameters. Five randomly selected fields at 100X magnification were examined for each section and analysed using the Automated Cell Counter tool (ImageJ software version 1.53j). Briefly, 8-bit images were created and segmented with a thresholding algorithm to eliminate the background and to highlight areas occupied by the nuclei. Collected data were transformed in binary form. Nuclei were automatically counted using previously set size and circularity parameters. Cell density is expressed per mm^2^ of tissue.

### Statistical Analysis

Statistical analysis was performed using two-way ANOVA (SPSS 19.1; IBM). Data are presented as the mean ± the standard deviation (SD). Differences of p ≤ 0.05 were considered significant and are indicated with different superscripts.

## Results

### miR-200b/c Exposure Induces High Plasticity and Erase Signs of Senescence in Fibroblasts Isolated From Aged Individuals

**Fig. 1:**
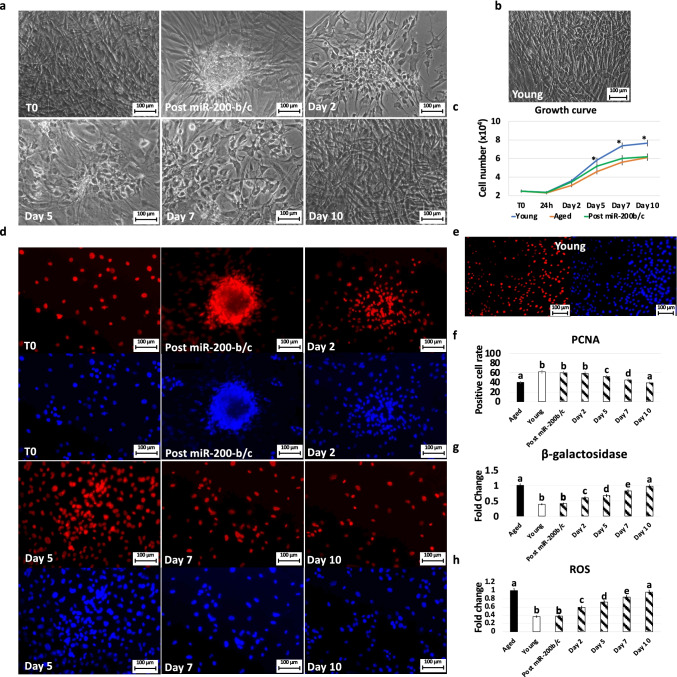
Morphology, growth curve, PCNA immunostaining/positive cell rate, β-GAL and ROS activity quantification in miR-200b/c treated aged fibroblasts cultured in 2D systems. **a** After miR-200b/c exposure, fibroblasts isolated from aged individuals loss their typical elongated shape (T0) and become smaller in size with granular, vacuolated cytoplasm, larger nuclei and form distinguishable aggregates (Post miR-200b/c). When fibroblasts returned to FCM progressively reverted to the original phenotype (Day 2, day 5 and day 7) and, by day 10, exhibited small central nuclei and an elongated spindle shape. Scale bars: 100 μm (**b**) Fibroblasts isolated from Young individuals displayed the typical elongated morphology. Scale bars: 100 μm (**c**) miR-200b/c exposed cells (Post miR-200b/c, green line) increment proliferation rate immediately after treatment and gradually return comparable to untreated aged cells (Aged, orange line). Data are expressed as the mean. Error bars represent the standard error of the mean (SEM). *p < 0.05. **d** PCNA (upper panels) and DAPI (lower panels) immunocytochemical staining in untreated fibroblasts isolated from aged individuals (T0), at the end of 48 h miR-200b/c exposure (Post miR-200b/c), at day 2, 5, 7, 10 of culture in 2D systems. Scale bars: 100 μm. **e** PCNA (left panel) and DAPI (right panel) immunocytochemical staining in untreated fibroblasts isolated from Young individuals. Scale bars: 100 μm. **f** PCNA quantitative evaluation in untreated fibroblasts isolated from aged (Aged, black bar) and young individuals (Young, white bars), at the end of 48 h miR-200b/c exposure (Post miR-200b/c) and at day 2, 5, 7, and 10 of culture in 2d systems. Data are expressed as the mean. Error bars represent the standard error of the mean (SEM). Different superscripts indicate *p* < 0.05. **g** β-GAL activity and (**h**) ROS levels in untreated fibroblasts isolated from aged (Aged, black bars) and young individuals (Young, white bars), at the end of 48 h miR-200b/c exposure (Post miR-200b/c) and at day 2, 5, 7, and 10 of culture in 2d systems. Data are expressed as the mean. Error bars represent the standard error of the mean (SEM). *p < 0.05

After miR-200b/c exposure, fibroblasts isolated from aged patients showed considerable phenotype changes. More in detail, the typical elongated shape, visible in untreated fibroblasts (T0, Fig. 1a), was replaced by a stem-cell-like morphology with cells smaller in size, granular and vacuolated cytoplasm, and larger nuclei. In addition, miR-2020b/c treated fibroblasts rearranged in a reticular pattern and tended to form distinguishable aggregates (Post miR-200b/c, Fig. 1a). Morphological changes were also accompanied by a significant increment in cell proliferation (Post miR-200b/c, Fig. 1c). In agreement with this, Post miR-200b/c aged cells showed a growth curve comparable to that of untreated fibroblasts isolated from young individuals (Young, Fig. 1c). PCNA immunocytochemical analysis confirmed these data (Fig. 1d) and, in particular, its quantitative evaluation demonstrated a significantly higher PCNA positive cell rate after miR-200b/c exposure (Post miR-200b/c, Fig. 1f).

These changes were paralleled by a significant decrease in β-galactosidase (β-GAL) activity (Post miR-200b/c, Fig. 1g) and reactive oxygen species (ROS) levels (Post miR-200b/c, Fig. 1h), with values post-treatment comparable to those young cells (Young, Fig. 1g and h).

**Fig. 2:**
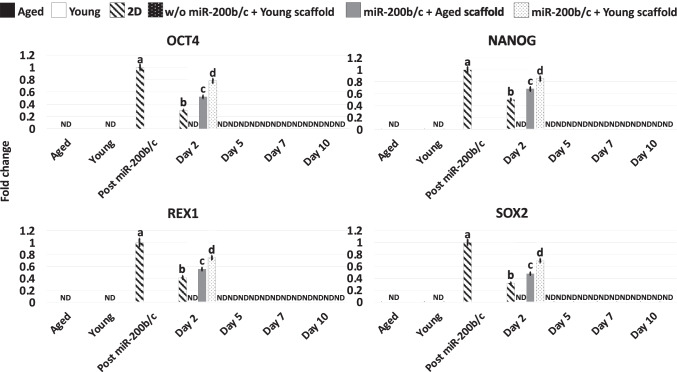
Gene expression levels of pluripotency-related (OCT4, NANOG, REX1, SOX2) genes in untreated fibroblasts isolated from aged (Aged, black bars) and young individuals (Young, white bars), at the end of miR-200b/c exposure, in miR-200b/c treated aged fibroblasts cultured in 2D systems (2D, striped bars), untreated aged fibroblasts cultured onto young 3D ECM-based bio-scaffolds (w/o miR-200b/c + Young scaffold, dotted black bars), miR-200b/c treated aged fibroblasts cultured onto aged 3D ECM-based bio-scaffolds (miR-200b/c + Aged scaffold, grey bars), and miR-200b/c treated aged fibroblasts cultured onto young 3D ECM-based bio-scaffolds (miR-200b/c + Young scaffold, dotted white bars). Data are expressed as the mean. Error bars represent the standard error of the mean (SEM). Different superscripts indicate *p* < 0.05

**Fig. 3:**
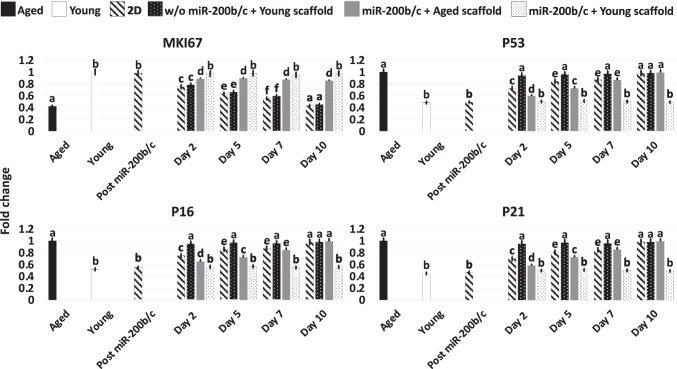
Gene expression levels of mitochondrial activity (TFAM, PDHA1, COX4I1) and reactive oxygen species modulator (ROMO1) genes in untreated fibroblasts isolated from aged (Aged, black bars) and young individuals (Young, white bars), at the end of miR-200b/c exposure, in miR-200b/c treated aged fibroblasts cultured in 2D systems (2D, striped bars), untreated aged fibroblasts cultured onto young 3D ECM-based bio-scaffolds (w/o miR-200b/c + Young scaffold, dotted black bars), miR-200b/c treated aged fibroblasts cultured onto aged 3D ECM-based bio-scaffolds (miR-200b/c + Aged scaffold, grey bars), and miR-200b/c treated aged fibroblasts cultured onto young 3D ECM-based bio-scaffolds (miR-200b/c + Young scaffold, dotted white bars). Data are expressed as the mean. Error bars represent the standard error of the mean (SEM). Different superscripts indicate p < 0.05

**Fig. 4:**
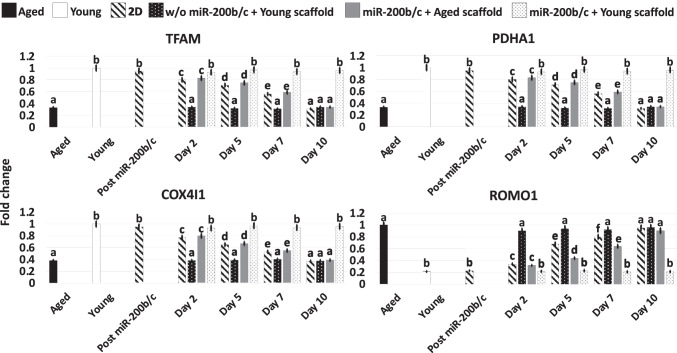
Gene expression levels of cell proliferation (MKI67) and senescence-related (P53, P16, P21) genes in untreated fibroblasts isolated from aged (Aged, black bars) and young individuals (Young, white bars), at the end of miR-200b/c exposure, in miR-200b/c treated aged fibroblasts cultured in 2D systems (2D, striped bars), untreated aged fibroblasts cultured onto young 3D ECM-based bio-scaffolds (w/o miR-200b/c + Young scaffold, dotted black bars), miR-200b/c treated aged fibroblasts cultured onto aged 3D ECM-based bio-scaffolds (miR-200b/c + Aged scaffold, grey bars), and miR-200b/c treated aged fibroblasts cultured onto young 3D ECM-based bio-scaffolds (miR-200b/c + Young scaffold, dotted white bars). Data are expressed as the mean. Error bars represent the standard error of the mean (SEM). Different superscripts indicate *p* < 0.05

Gene expression analysis were consistent with the morphological observations and indicated the onset of the pluripotency-related genes OCT4, NANOG, REX1, and SOX2 (Post miR200b/c, Fig. 2), which were originally undetectable in untreated fibroblasts (Aged, Young; Fig. 2). Furthermore, Post miR-200b/c significantly increased their expression of the cell proliferation marker MKI67 (Fig. 4), and the mitochondrial activity-related genes TFAM, PDHA1, and COX4I1 (Fig. 3), picking to values comparable to young cells (Young; Fig. 3). Transcription levels of the reactive oxygen species modulator ROMO1 and of the senescence-related markers, P53, P16 and P21, significantly decreased to levels distinctly of young cells (Young; Fig. 4).

### miR-200b/c Treated Fibroblasts Cultured in 2D Systems Revert to Their Original Phenotype

Fibroblasts returned to FCM, after removal of miR-200b/c, progressively reverted to the original phenotype (Day 2, day 5 and day 7; Fig. 1a) and, by day 10, exhibited small central nuclei and an elongated spindle shape, comparable to those of untreated aged fibroblasts (T0, Fig. 1a). The morphological changes were also paralleled by a decrement in cell proliferation (Fig. 1c). This was also supported by PCNA positive cell rate values that slowly decreased, returning statistically comparable to those of aged fibroblasts by day 10 of culture (Fig. 1d and f). Consistently, β-GAL activity (Fig. 1g) and ROS levels (Fig. 1h) gradually incremented during the culture period, up to day 10, when the values detected were statistically comparable to those observed in untreated aged cells (Aged).

Molecular analysis confirmed these data, with pluripotency-related gene OCT4, NANOG, REX1, and SOX2 expression undetectable by day 5 of culture in FCM (Fig. 2). Moreover, by day 10 ROMO1 (Fig. 3), P53, P16, and P21 (Fig. 4) gene transcription returned statistically comparable to that of untreated aged cells (Aged). By day 10 MKI67 (Fig. 4), TFAM, PDHA1, COX4I1 genes (Fig. 3) displayed expression levels comparable to those detected in untreated aged cells (Aged).

### miR-200b/c Treated Fibroblasts Repopulate 3D ECM-based Bio-scaffolds, but Maintain a Rejuvenated Phenotype Only on Young ECM

**Fig. 5:**
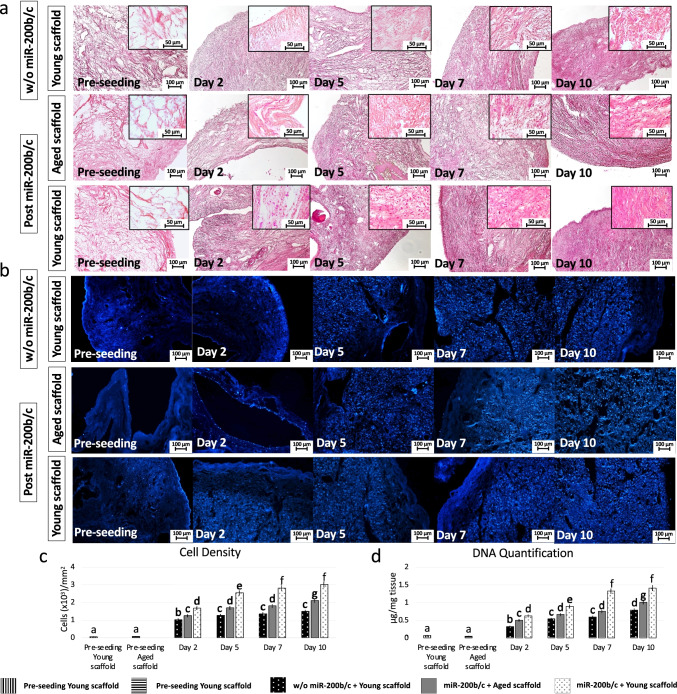
H&E, DAPI staining, cell density and DNA quantification in miR-200b/c treated aged fibroblasts cultured onto 3D ECM-based bio-scaffolds. **a** H & E and (**b**) DAPI staining before cell seeding (Pre-seeding), in untreated aged fibroblasts (w/o miR-200b/c) plated onto young 3D ECM-based bio-scaffolds (Young scaffold) and in miR-200b/c treated aged fibroblasts (Post miR-200b/c) plated onto aged (Aged scaffold) or onto young 3D ECM-based bio-scaffolds (Young scaffold) at day 2, 5, 7, and 10 of culture. Scale bars: 100 μm. Insert scale bars: 50 μm. **c** Cell density analyses before cell seeding in aged (Pre-seeding aged scaffold, vertical stripped bars) and young scaffolds (Pre-seeding young scaffold, horizontal stripped bars), in untreated aged fibroblasts plated onto young 3D ECM-based bio-scaffolds (w/o miR-200b/c + Young scaffold, dotted black bars) and in miR-200b/c treated aged fibroblasts plated onto aged (miR-200b/c + Aged scaffold, grey bars) or onto young 3D ECM-based bio-scaffolds (Young scaffold) at day 2, 5, 7, and 10 of culture. Data are expressed as the mean. Error bars represent the standard error of the mean (SEM). Different superscripts indicate *p* < 0.05. **d** DNA quantification before cell seeding in aged (Pre-seeding aged scaffold, vertical stripped bars) and young scaffolds (Pre-seeding young scaffold, horizontal stripped bars), in untreated aged fibroblasts plated onto young 3D ECM-based bio-scaffolds (w/o miR-200b/c + Young scaffold, dotted black bars) and in miR-200b/c treated aged fibroblasts plated onto aged (miR-200b/c + Aged scaffold, grey bars) or onto young 3D ECM-based bio-scaffolds (Young scaffold) at day 2, 5, 7, and 10 of culture. Data are expressed as the mean. Error bars represent the standard error of the mean (SEM). Different superscripts indicate *p* < 0.05

H & E (Fig. 5a) and DAPI staining (Fig. 5b) demonstrated a comparable repopulating ability in miR-200b/c treated cells as well as in untreated fibroblasts (w/o miR-200b/c), indicating that miR-200b/c treatment does not affect cell engrafting ability. Moreover, cell density analyses showed an increasing number of cells along the culture period in all the experimental groups (Fig. 5c). Nevertheless, significant differences among the experimental groups were detected. Specifically, starting from day 2 of co-culture and onward, cell number was significantly higher in young decellularized bio-scaffolds repopulated with miR-200b/c exposed fibroblasts (miR-200b/c + Young scaffold) compared to miR-200b/c + Aged scaffold and the control group (w/o miR-200b/c + Young scaffold, Fig. 5c). These observations were also confirmed by DNA quantification (Fig. 5d).

**Fig. 6:**
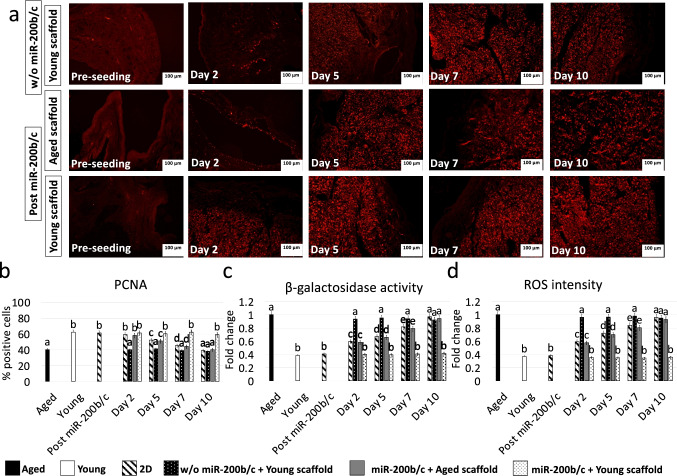
PCNA immunostaining/positive cell rate, β-GAL and ROS activity quantification in miR-200b/c treated aged fibroblasts cultured onto 3D ECM-based bio-scaffolds. **a** PCNA immunocytochemical staining before cell seeding (Pre-seeding), in untreated aged fibroblasts (w/o miR-200b/c) plated onto young 3D ECM-based bio-scaffolds (Young scaffold) and in miR-200b/c treated aged fibroblasts (Post miR-200b/c) plated onto aged (Aged scaffold) or onto young 3D ECM-based bio-scaffolds (Young scaffold) at day 2, 5, 7, and 10 of culture. Scale bars: 100 μm. **b** PCNA quantitative evaluation in untreated fibroblasts isolated from aged (Aged, black bars) and young individuals (Young, white bars), at the end of 48 h miR-200b/c exposure (Post miR-200b/c), in untreated aged fibroblasts plated onto young 3D ECM-based bio-scaffolds (w/o miR-200b/c + Young scaffold, dotted black bars) and in miR-200b/c treated aged fibroblasts plated onto aged (miR-200b/c + Aged scaffold, grey bars) or onto young 3D ECM-based bio-scaffolds (Young scaffold) at day 2, 5, 7, and 10 of culture. Data are expressed as the mean. Error bars represent the standard error of the mean (SEM). Different superscripts indicate *p* < 0.05. **e** β-GAL activity and **f** ROS levels in untreated fibroblasts isolated from aged (Aged, black bars) and young individuals (Young, white bars), at the end of 48 h miR-200b/c exposure (Post miR-200b/c), in untreated aged fibroblasts plated onto young 3D ECM-based bio-scaffolds (w/o miR-200b/c + Young scaffold, dotted black bars) and in miR-200b/c treated aged fibroblasts plated onto aged (miR-200b/c + Aged scaffold, grey bars) or onto young 3D ECM-based bio-scaffolds (Young scaffold) at day 2, 5, 7, and 10 of culture. Data are expressed as the mean. Error bars represent the standard error of the mean (SEM). Different superscripts indicate *p* < 0.05

PCNA staining reveled immuno-positivity in all groups considered (Fig. 6a). However, significantly high number of PCNA positive cells were scored in young scaffolds repopulated with miR-200b/c treated fibroblasts (miR-200b/c + Young scaffold, Fig. 6b). This is coherent with β-GAL (Fig. 6c) and ROS (Fig. 6d).

Gene panel evaluation indicated the loss of the pluripotency-related genes by day 5 of co-culture, as expected and previously demonstrated [15–21, 33]. In addition, transcription of MKI67, TFAM, PDHA1, COX4I1, ROMO1, P53, P16, and P21 genes (Figs. 3 and 4) was maintained at levels statistically comparable to those distinctive of young cells only in miR-200b/c + Young scaffold (Figs. 3 and 4).

## Discussion

In the present study, we provide evidence that the use of miR-200b/c induces a transient high plasticity state and ameliorates cellular and physiological hallmarks of aging in senescent cells. In addition, we demonstrate that the rejuvenated phenotype is stably maintained when exposure to miR-200b/c is coupled with engrafting onto young ECM-based bio-scaffolds.

More in detail, exposure to miR-200b/c induces significant changes in fibroblasts isolated from aged patients that acquire morphological and molecular features distinctive of pluripotent cells. Fibroblast standard elongated shape is replaced by a round or oval one. Cells become smaller in size with granular, vacuolated cytoplasm and rearrange in distinguishable aggregates. All these aspects closely resemble those previously described for human ESCs [34] and iPSCs [35], which show a typical round morphology and form compact colonies, with distinct borders and well-defined edges [34–38]. In addition, senescent cells exposed to miR-200b/c display larger nuclei with less cytoplasm, an aspect usually correlated to the relaxed and accessible chromatin structure typically exhibited by pluripotent cells [39–42]. All these observations suggest that the use of miR-200b/c may encourage the appearance of morphological properties previously described in both native and induced high plasticity cells, which is also supported by the molecular analyses performed, demonstrating the onset of the pluripotency-related genes, OCT4, NANOG, REX1, and SOX2, in response to miR-200b/c exposure. Overall, these data confirm and further expand previous studies indicating that overexpression of miR-200 supports Nanog active transcription and ESC self-renewal, while inhibiting embryoid body formation and repressing the expression of ectoderm, endoderm, and mesoderm markers [43, 44].

Interestingly, the morphological changes described above are paralleled by significant reduction of aging hallmarks. In particular, miR-200b/c exposure causes an increment in cellular growth curve values, in proliferation rates and PCNA immune-positive cell number. These results suggest cell-cycle re-activation and the reestablishment of a robust cell division in senescent cells challenged with miR-200b/c. Consistent with this, recent pilot works described the possibility to achieve a young phenotype after restoration of a vigorous cell growth in non-dividing quiescent and senescent cells [45, 46], pointing to a scenario where proliferation is an essential requirement for cellular rejuvenation [47].

Reduction of senescence-associated markers after miR-200b/c exposure is also demonstrated by a significant decrease in β-GAL and ROS activities and is in line with a recent study, showing miR-200 treatment ability to induce a decrement in β-GAL levels as well as in P53 and P21 gene transcription [48]. Consistent with this, our molecular data indicate that miR-200b/c treated cells display a downregulation of P53 and P21 as well as P16 genes. In addition, physiological signs of aging are damped by reducing the oxidative stress (ROMO1), promoting cell proliferation (MKI67) and increasing mitochondrial activity (TFAM, PDHA1, and COX4I1). Altogether these observations confirm the key role played by the miR-200 family, not only to promote and maintain high plasticity [29, 43, 49], but also to reduce signs of cellular senescence and to encourage the acquisition of a young phenotype. It is however important to note that when miR-200b/c are removed from cultures and cells are returned to FCM, they gradually revert to their original phenotype, exhibiting all morphological and molecular signs distinctive of aged cells by day 10 of culture. Specifically, cells increase their size, appear longer with elongated shape, and decrease both their proliferation indexes and PCNA positive rates. The reacquisition of a senescent phenotype is also confirmed by an increased β-GAL and ROS activities and by changes in the transcription levels of MKI67, TFAM, PDHA1, COX4I1, ROMO1, P53, P16, and P21 genes, that returned statistically comparable to those of untreated aged cells. In our understanding, these results demonstrate not only an evident cell rejuvenation induced by miR-200b/c treatment, but also that this effect is transient and reversible.

In contrast, the rejuvenated state appears to be stably retained when cells are engrafted onto young 3D ECM-based bio-scaffolds. Indeed, following miR-200b/c removal, cells adhere and colonize the bio-scaffolds stably maintaining all the hallmarks typical of young cells, namely high number of PCNA positive cells, low values of β-GAL and ROS activities, high transcription levels for the genes involved in cell proliferation (MKI67), mitochondrial activity (TFAM, PDHA1, and COX4I1) and low transcription for the genes associated to cell senescence, such as ROMO1, P53, P16, and P21. These results point to the possibility that a young microenvironment may exert a supporting effect to stably maintain the rejuvenated phenotype induced by miR-200b/c treatment, which would otherwise be transient and quickly lost.

## Conclusion

In conclusion, the data presented in this manuscript show that multiple factors cooperate to control a unique program, driving the cell clock. In particular, molecular mechanisms regulated by miR200 are directly involved in erasing cellular senescence, however, an adequate microenvironment is required to stabilize the rejuvenating effects, suggesting the involvement of synergistic interactions among molecular effectors and bio-scaffold-derived mechanical cues. It is clear that ovarian ECM creates a unique microenvironment deriving from the combination of its complex microanatomy and heterogenous architecture that may provide different mechanotransducive cues, influencing cell proliferation and function as well as resulting in the activation of local intracellular pathways.

Altogether, the model here developed represents, in our understanding, a useful tool to better characterize these complex regulations and to allow a fine dissection of the multiple and concurring biochemical and biomechanical stimuli driving the process.

## Data Availability

The data presented in this study are available on request from the corresponding author.
